# Nutraceutic Potential of Bioactive Compounds of *Eugenia dysenterica* DC in Metabolic Alterations

**DOI:** 10.3390/molecules27082477

**Published:** 2022-04-12

**Authors:** Lidiani Figueiredo Santana, Sandramara Sasso, Diana Figueiredo Santana Aquino, Karine de Cássia Freitas, Rita de Cássia Avellaneda Guimarães, Arnildo Pott, Valter Aragão do Nascimento, Danielle Bogo, Patrícia de Oliveira Figueiredo, Priscila Aiko Hiane

**Affiliations:** 1 Graduate Program in Health and Development in the Central-West Region of Brazil, Federal University of Mato Grosso do Sul—UFMS, Campo Grande 79070-900, Brazil; lidi_lfs@hotmail.com (L.F.S.); sandramara_sasso@hotmail.com (S.S.); rita.guimaraes@ufms.br (R.d.C.A.G.); aragao60@hotmail.com (V.A.d.N.); daniellebogo@hotmail.com (D.B.); priscila.hiane@ufms.br (P.A.H.); 2 Higher Level Technician, Personnel Development Division, State University of Mato Grosso do Sul—UEMS, Dourados 79804-970, Brazil; di_fsa@yahoo.com.br; 3 Institute of Biosciences, Federal University of Mato Grosso do Sul—UFMS, Campo Grande 79079-900, Brazil; arnildo.pott@gmail.com; 4 Laboratory Pronabio (Bioactive Natural Products)-Chemistry Institute, Federal University of Mato Grosso do Sul—UFMS, Campo Grande 79074-460, Brazil; patricia.oliveira@ufms.br

**Keywords:** Brazilian fruit, chronic diseases, inflammation mediators

## Abstract

The fruit and leaves of *Eugenia dysenterica* DC., locally known as *cagaita*, are rich in antioxidant glycosylated quercetin derivatives and phenolic compounds that have beneficial effects on diabetes mellitus, hypertension and general inflammation. We conducted a literature search to investigate the nutraceutical potentials of these phenolic compounds for treating obesity, diabetes mellitus and intestinal inflammatory disease. The phenolic compounds in *E. dysenterica* have demonstrated effects on carbohydrate metabolism, which can prevent the development of these chronic diseases and reduce LDL (low-density lipoprotein) cholesterol and hypertension. *E. dysenterica* also improves intestinal motility and microbiota and protects gastric mucosa, thereby preventing inflammation. However, studies are necessary to identify the mechanism by which *E. dysenterica* nutraceutical compounds act on such pathological processes to support future research.

## 1. Introduction

Myrtaceae stands out as one of the most important families of tropical and subtropical flora, distributed over all habitable continents [1], with 130 genera and 4000 species. Considered the most diverse family in the neotropics and some humid forests, in Brazil, it accounts for 10–15% of the vegetation from Amazonia to the Rio Grande do Sul. It is the third best-represented family in the Brazilian Cerrado [2], encompassing ca. 23 genera and approximately 1000 species.

The genus *Eugenia* is widely known as the target of study for its anti-hyperglycemic potential. For example, *E. uniflora* (Brazilian cherry) is used as an antipyretic, anti-inflammatory and anti-hyperglycemic, and has been investigated for the treatment of digestive diseases [3]. Species of another genus, such as *Syzygiumcumini* (*E. jambolana*, Malabar plum), havedemonstrated potential as an anti-hyperglycemic and anti-hypoglycemia due to fractions rich in flavonoids that are capable of modulating enzymes that metabolize carbohydrates and lipids and attenuate the differentiation of adipocytes in vitro [4,5]. The fruit and leaves of *Syzygiummalaccense* (*E. malaccensis*, Malay apple) are used as an astringent, appetizer, diuretic, anti-inflammatory and antidiabetic and in the treatment of anemia [3].

*Eugenia dysenterica* DC., popularly known as cagaita, is another Myrtaceae fruit species, widely used to treat diarrhea, diabetes mellitus, jaundice, renal infections and constipation [6,7]. The laxative effect comes mainly from ripe fruit at initial fermentation [7] and is caused by a specific peptide capable of increasing intestinal motility [8] and inhibiting intestinal secretion and absorption [9].

Moreover, its leaves have antidiarrheic properties and have been reported in the treatment of diabetes [10]. Studies have also identified an antifungal activity due to the essential oil in the leaves, which are rich in sesquiterpenes (e.g., beta-caryophyllene and alpha-humulene) and monoterpenes (e.g., limonen and alpha-thujone) [11]. Stand out salutary compounds are the antioxidant vitamin C [12] and phenols (flavonoids and tannins) [13] that have proven benefits for treating hypertension and general inflammation [14,15,16].

*E. dysenterica* is a beneficial species, and scientific studies have demonstrated the biological and medicinal features of different parts of the plant. However, few studies have reported the therapeutical potential for metabolic dysfunctions through specific experimental models of obesity or general inflammation. Therefore, our review investigated the nutritional value and the bioactive compounds of *E. dysenterica*, existing medicinal uses, and possible metabolic and anti-inflammatory applications.

## 2. Nutritional Properties of *Eugenia dysenterica*

### Chemical and Phytochemical Composition

**Figure 1:**
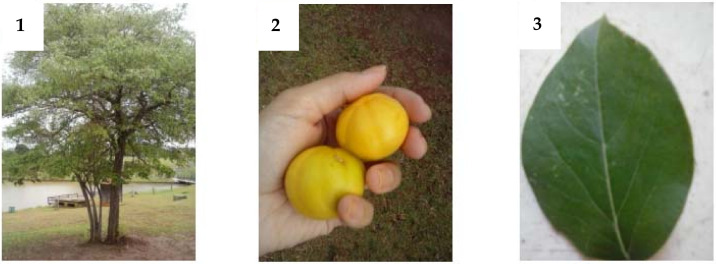
*E. dysenterica* DC. (**1**) tree, (**2**) fruit and (**3**) leaf. (Photos: L. F. Santana.).

Belonging to the familyMyrtaceae and genus *Eugenia,* thespecies *Eugenia dysenterica*is a tree of medium height (4–10 m) with tortuous branches and trunks. New leaves are membranaceus; adult leaves are coriaceous and are glabrous on both faces. At flowering, it forms fasciculate panicles, delicate white flowers with four petals, a calyx of four lacinia ovate and ciliated, and the fruit is globose: succulent light-yellow berries that have a pleasant and slightly acidic taste. Its seeds are ellipsoid and flattened [17] (Figure 1).

The fruit of *E. dysenterica* has a high pulp yield (86.43 ± 4.23%), and 100 g of pulp has low energetic value (30.33 kcal) [17], including 1.14 g of proteins, 0.57 of fats and 6.33 g of carbohydrates. [18] (Table 1).

Regarding the profile of fatty acids, *E. dysenterica* is one of the fruits with the highest percentage of polyunsaturated fatty acids, with linoleic acid (10.5%) and linolenic acid (11.86%) representing the major fractions [20,21].

In 100 g of seeds, there are expressive contents of carbohydrates (14.85 g) and low amounts of proteins and lipids (0.59 and 0.26 g, respectively), with a concentration of unsaturated fat of 62.3%, of which 21.18% are monounsaturated acids and 41.15% polyunsaturated acids. The main component is α-linoleic acid (3.05%). Among the monounsaturated and polyunsaturated acids, oleic acid (20.17%) and linoleic acid (38.11%) stand out as having the highest quantities [19].

The minerals identified in the pulp are calcium and iron, representing 8.0 and 0.02 mg per 100 g of pulp, respectively [22]. The leaves were observed to contain zinc (21.40 ppm), phosphorous (0.14%), potassium (1.20%), calcium (0.84%), magnesium (0.28%), sulphur (0.06%), boron (32.50 ppm), copper (15 ppm), manganese (163 ppm) and iron (145 ppm). In addition, in 100 g of pulp, there are high contents of vitamin C (24.53 mg) (ascorbic and dehydroascorbic acids), folates (25.74 μg) and carotenoids (0.77 mg), found in seeds and leaves, as well as identified tocopherols, lycopene and tannins [23] (Table 2).

The fruit of *E. dysenterica* is tasty and rich in nutritional substances, such as ascorbic acid (vitamin C), organic acids (acetic, lactic, malic, tartaric, succinic and citric), tannins, carotenoids (α-carotene, β-carotene, β-cryptoxanthin and lycopene) and vitamin E (α-, β-, γ- and δ-tocopherol and tocotrienol) [25].

The fruit pulp is also considered a source of phenols, containing derivatives of quercetin and kaempferol, ellagic acid, ellagitannins and proanthocyanidins [26]. High phenolic compounds (98 mg/equivalent of gallic acid (GAE)/g) and proanthocyanidins (42 g/g) showed low to middle antioxidant activity, in the range of 5.8 to 26.5% for DPPH, 13.9 to 43.1% for FRAP, and 13.6 to 36.5% for ORAC (Table 3) [27].

**Figure 2:**
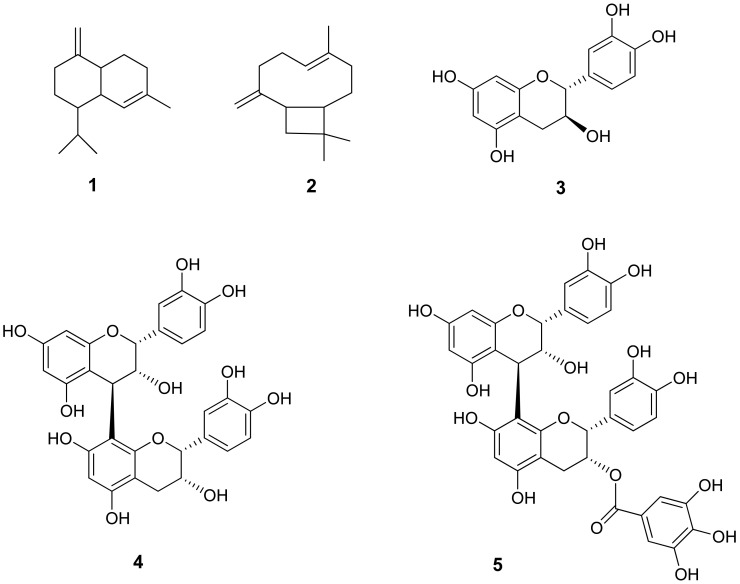
Structures of major components isolated from *E. dysenterica*, including γ-cadinene (**1**), β-caryophyllene (**2**) and polyphenolic compounds including catechin (**3**), procyanidin-B1 (**4**), and dimeric procyanidin gallate (**5**) [30]. 3. Therapeutic Properties of *E. dysenterica*.

The therapeutical properties of *E. dysenterica* are attributed to its bioactive compounds, mainly phenolic acids, flavonoids, anthocyanins and organic acids present in fruits [28], and polyphenols [29], γ-cadinene, β-caryophyllene and δ-cadinene present in leaves (Figure 2) [30].

## 3. Therapeutic Properties of *E. dysenterica*

### 3.1. Effects of E. dysenterica on Oxidative Stress

Oxidation is a metabolic process resulting from the production of energy for cell activities;however, oxygen metabolism in living cells concomitantly produces free radicals, and these, when not controlled, can cause extensive damage [17]. Oxidative stress has been linked to the development of many chronic and degenerative diseases, including cancer, heart disease, and degenerative diseases such as Alzheimer’s.Furthermore, it is involved in the aging process [18,19].

Phenolic compounds, in particular polyphenols, have antioxidant properties that are recognized for reducing the viability of adipocytes and proliferation of pre-adipocytes; they also suppress the differentiation of adipocytes and buildup of triglyceride. They also stimulate lipolysis and β-oxidation of fatty acids and reduce inflammation and oxidative stress, which supports the anti-inflammation associated with treating obesity and its metabolic disorders [29].

Polyphenols exert their additive and or synergic effects through one or more signaling and transcription mechanisms, including those mediated by nuclear factor kappa B (NF-κb), AMP-activated protein kinase (AMPK), PPARγ and peroxisome proliferator-activated receptor gamma coactivator 1-alpha (Figure 2) (PGC-1α) [29].

Vitamin E is liposoluble, existing in two main subgroups: tocopherol and tocotrienol. Evidence has demonstrated that both tocopherol and tocotrienol have relevant metabolic effects, such as anti-obesity, anti-hypercholesterolemic, anti-diabetic and anti-hypertensive effects [31].

Citric acid, also present in *E. dysenterica* fruit, has an important antioxidant effect that can regulate oxidative stress in obesity, diminish lipid peroxidation and inflammation, reduce cellular degranulation and attenuate the liberation of inflammatory compounds such as myeloperoxidase, elastase, interleukin and platelet factor-4. In animal studies, citric acid at 1.0–2.0g/kg of animal body weight, given via gavage, was able to diminish the peroxidation and inflammation of brain lipids, liver damage, and fragmentation of DNA (deoxyribonucleic acid); it also improved the relaxation and contraction of vascular endothelium, blood clotting, platelet aggregation, and immune function [31].

Tartaric acid present in the fruit of *E. dysenterica* and many other species, mainly grapes, is obtained as a solid during fermentation. Currently, no therapeutic or pharmacological uses have been attributed to it because it is mostly used as a natural acidifier and preservative. Malic acid, which is frequently found in cosmetics in the form of 1.0% aerosol, improved xerostomia induced by anti-hypertensives and stimulated saliva production. It is known for presenting a significant antiproliferative effect in HaCaT cells (human keratinocytes), inhibiting the progression of the cell cycle in G0/G1 and stimulating the induction of programmed cell death [32].

### 3.2. Effects of E. dysenterica on Diabetes and Inflammation

Proper regulation and management of energy are fundamental to cell and organism survival. The immune response protects cellular organization by recognizing and eliminating pathogenic signals and safeguarding tissue homeostasis. Proper maintenance of this delicate balance has important implications for obesity, diabetes and other chronic non-communicable diseases [33].

Diabetes mellitus is a set of metabolic diseases characterized by hyperglycemia resulting from defects in pancreatic secretion or insulin resistance [34], resulting in a glucose metabolism imbalance that can lead to increased insulin production or decreased glucose uptake by insulin-dependent tissues [35]. Currently, diabetes is classified into type 1, type 2, and gestational hyperglycemia [36].

A serious complication of diabetes is oxidative stress caused by the generation of reactive oxygen species (ROS) [37,38]. In this context, the search for natural compounds with beneficial health effects is currently gaining special attention for mitigating or preventing chronic non-communicable diseases [39,40,41].

*E. dysenterica* fruit and leaves are used as an alternative medicine by local communities to treat diarrhea, diabetes, and jaundice. Phenolic compounds from the fruit have demonstrated in vitro antioxidant potential and inhibitory actions toward the activity of enzymes involved in carbohydrate metabolism, preventing the development of obesity and type 2 diabetes [28,42,43].

Polyphenols, widely known for their antioxidant and anti-inflammatory properties, can be used in a strategy to deal with obesity by decreasing oxidative stress and levels of (ROS), which contribute to insulin resistance and have an extensive association with markers of oxidative stress, obesity and diabetes [29].

The fruit pulp showed beneficial effects on carbohydrate metabolism, withhigher activity on the enzymes α-amylase and α-glycosidase [44]. A study showed that the effects of reducing postprandial blood glucose in dysglycemic individuals after consumption of clarified *E. dysenterica* juice were probably related to the action of polyphenols, which reduced the amount of glucose absorbed since no fibers in the juice were detected. In that test, clarification was achieved through the homogenization of fresh fruit or commercial frozen pulp, followed by centrifugation in a Sorvall refrigerated centrifuge model RC-5C, rotor type GSA (22.770 g/40 min, 4 °C) [45,46].

For obesity treatment, leaf extract did not alter weight gain during the evaluation period, but it demonstrated the ability to act as an adjunct in initial weight loss by helping to reduce food intake [47].

Another study evaluated the effects of *E. dysenterica*on mice with streptozotocin-induced diabetes and found that the ethanol extract of the leaves did not affect blood glucose levels, but it did improve the lipid profile, which shows potential for treating dyslipidemia [29].

The essential oil of *E. dysenterica* leaves has been shown to stimulate in vitro skin cell migration and inhibit nitric oxide, suggesting an anti-inflammatory effect and the promotion of angiogenesis in vivo [48].

Some studies verified that the abundant organic acids with different and complex compositions (e.g., tartaric, malic, lactic and citric acids) reduced hyperglycemia and hyperinsulinemia through delayed gastric emptying, suppression of hepatic glucose production, increased use of glucose, positive regulation of vasodilation mediated by flux, and facilitated secretion of insulin. In addition, these acids reduced hyperlipidemia by inhibiting the metabolic pathways of cholesterogenesis and lipogenesis in the liver. This was made possible by activating AMPK, an inhibitor of fatty acid–sterol synthesis and by inhibiting lipogenesis, which reduced the buildup of visceral fat [49,50].

The antioxidant effect of *E. dysenterica* demonstrated the ability to promote cytoprotection when associated with chlorhexidine gluconate, which can be used to help patients with oral disorders by preventing lipoperoxidation and acetylcholine activity [51,52].

In the literature, *E. dysenterica* was shown to have therapeutic potential for various types of metabolic dysfunctions, such as diabetes mellitus type 1 and 2, that led to alterations in both glycemic and lipidic metabolism and oxidative stress.

Antioxidants that protect against cell damage may support the treatment of various diseases, given that the excess of non-neutralized reactive species is related to the etiology and progression of hyperlipidemia, diabetes mellitus [41] and cancer [53].

To further these studies on the effects of *E. dysenterica* on metabolism, investigations into models of obesity and metabolic syndrome are needed to facilitate the search for new therapeutic approaches and a better understanding of the metabolic dysfunction associated with obesity.

### 3.3. Effects of Eugenia dysenterica on Obesity

Obesity is associated with metabolic diseases and chronic comorbidities, such as hypercholesterolemia, hypertriglyceridemia, insulin resistance, heart diseases, type 2 diabetes, atherosclerosis and cancer. The etiology of obesity is complex, resulting from the interaction of genetic and epigenetic environmental and emotional factors as well as lifestyle. Technically, obesity results from a positive energetic balance—more energy consumption than expenditure—caused by the occidental diet and a sedentary lifestyle [54].

Diet seems to play an essential role in the pathogenesis of obesity and, consequently, metabolic dysfunction. Alterations in the composition of macronutrients, such as increased amounts of saturated fat and carbohydrates, mainly refined sugars, help promote adipogenesis, insulin resistance and cellular dysfunction [55].

**Figure 3:**
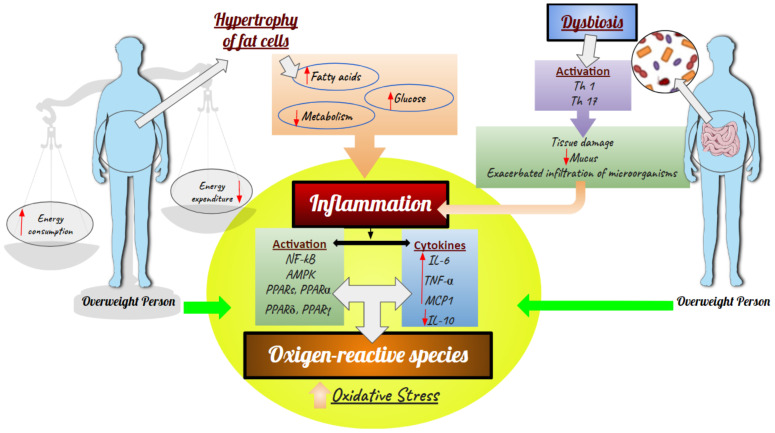
Effects of body fat buildup and dysbiosis in the inflammatory process and oxidative stress. NF-κB, AMPD–AMP-activated protein kinase, PPARs, IL-6, TNF-α, MCP1, IL-10, Th-lymphocytes T.

Overweight is characterized by increased deposition of white adipose tissue, leading to the overproduction of cytokines and metabolic alterations; that is, excess fatty acids and metabolites related to increased oxidation and oxidative stress are released into the blood stream, reducing the uptake and metabolism of glucose and activating pro-inflammatory paths through activation of NF-κB and increasing the basal activity of AMPK. Long-chain fatty acids also activate the proteins of peroxisome proliferator-activated receptors (PPARs), which regulate the metabolism of fats and carbohydrates through protein members of nuclear receptors, such as PPARα, PPARδandPPARγ (Figure 3) [29,54,55,56,57,58].

Furthermore, in response to the buildup of lipids, pro-inflammatory mediators induced by phenotypical changes in white adipose tissue are released, and these lead to organ dysfunction and low-degree chronic inflammation with high levels of pro-inflammatory cytokines (e.g., interleukin-6 (IL-6) and tumor necrosis factor-α (TNF-α)) and chemokines, such as monocyte chemoattraction protein1 (MCP1), which may promote migration of macrophages to adipose tissue, thereby increasing and liberating more cytokines. Similarly, reduced levels of interleukin 10 (IL-10), which regulates inflammatory responses, were observed. This reduction increases the activation of cytokines, which intensifies the inflammatory response, imbalance of antioxidant defenses and production of ROS (Figure 3) [29,54].

The breakdown of homeostasis in gut bacteria (dysbiosis) ruptures the intestinal barrier integrity, which is followed by increased permeability and consequent translocation of fragments of bacteria, mainly lipopolysaccharide (LPS) and uremic toxins, from the intestinal lumen into the bloodstream, inducing endotoxemia, also called low-degree inflammation. Moreover, the microbe-associated molecular pattern (MAMP) binds to Toll-like receptors (TLRs) like TLR4 and starts a pro-inflammatory cascade. This is further fueled by advanced glycation end products (AGEs), and other oxidative pathways are deeply involved in the metabolic dysregulation associated with obesity, glucose metabolism, insulin signaling and development of diabetes (Figure 3) [48,59].

Therefore, diminishing the levels of ROS is a relevant strategy for dealing with alterations related to obesity. Thus, the consumption of food rich in antioxidants and bioactive anti-inflammatory compounds, such as fatty acids n-3 (omega-3) and polyphenols, can diminish oxidative stress and increase the antioxidant capacity in adipose tissue. The thermogenesis and energy expenditure reduce inflammation and support the reduction of metabolic disorders.

In addition, the anti-obesity effects of diets rich in polyphenols can be attributed to their capacity in interacting, direct or indirectly, with adipose tissues (pre-adipocytes, adipose trunkcells and immunologic cells) [29]. Polyphenols are the most abundant phytochemicals in fruit and vegetables [47,59]. They represent a great variety of compounds, separated into classes according to their chemical structure: phenolic acids (hydroxybenzoic and hydroxycinnamic acids), flavonoids (flavonols, flavones, isoflavones, flavanones and anthocyanins), stilbenes, lignans and curcuminoids [60].

Evidence shows that the intake of fruit and a plant-based diet negatively correlates to the development of diabetes [54]. Such studies show that medicinal plants containing antioxidant compounds are popularly indicated for the treatment of obesity, including infusions and extracts, which are widely utilized for weight control. Therefore, it is pertinent to explore the potential of traditional medicinal plants for treating obese individuals beyond diminishing blood glucose levels and as biomarkers related to obesity [54,59,60].

The chemical characterization of *cagaita* (*E. dysenterica* DC.) revealed phytochemicals and micronutrients in various parts of the plant as previously described. In an animal obesity study showing the metabolic complications from ingesting a diet rich in fats and saccharose, male mice C57BL/6J received daily *E. dysenterica* extract rich in phenolic compounds by gavage at doses of 7 and 14 mg of GAE/kg of body weight. The main findings indicated that the extract attenuated body weight gain, adiposity, fasting hyperglycemia (but not postprandial glucose), hypertriglyceridemia, hypercholesterolemia, plasma antioxidant capacity and fecal triglyceride excretion in both treatments. The 7 mg dose also led to a significant reduction in hepatic triglycerides [26].

Reducing fats and carbohydrates is one dietary treatment option for obesity because it inhibits the enzymatic activity of pancreatic lipase. Drugs such as orlistat impede the action of lipases in the intestine and so diminish fat absorption, but an adverse effect is liposoluble vitamin and essential fat deficiency. However, phenolic compounds at 7 and 14 mg doses of GAE also inhibited pancreatic lipase in vitro and in vivo; that is, they can have anti-obesity properties. Nevertheless, those authors point out that studies are needed to evaluate such a hypothesis for *E. dysenterica* [26,61].

Furthermore, the polyphenol-rich *E. dysenterica* extract attenuated hepatic gluconeogenesis and inflammation caused by the expression of TNF-α and NF-κB [26]. In the liver, the Kupffer cells produce several inflammatory mediators (cytokines, prostaglandins and ROS) that are involved in the development of obesity-related comorbidities such as hepatic insulin resistance [62]. Therefore, those authors supposed that the improved glucose homeostasis and metabolic parameters induced by the extract were attributable, at least in part, to a function optimization [26].

In another study, healthy individuals consumed seven test meals, with a one-week interval in between, consisting of 50 g of white bread and 300 mL of water (control) or the fruit juices of *Campomanesiaphaea* (flying saucer fruit, *cambuci*), *E. dysenterica*, *Passiflora tenuifila* (garlic passion fruit), *Theobroma grandiflorum* (*cupuaçu*), *Myrciariadubia* (*camu-camu*) and *M. cauliflora* (*jabuticaba*). At the end of the study, the main finding was that the juices rich in polyphenols reduced the blood glucose levels sharply after ingesting meals rich in carbohydrates. The highest reducer response of glucose serum concentrations was observed after consumption of *E. dysenterica* (64%), followed by *C. phaea* (36%), *M. dubia* (31%), juice of *M. cauliflora* (24%), *T. grandiflorum* (20%) and *P. tenuifila* (11%), in comparison with control. The hypoglycemia actions were partially attributed to phytochemicals, such as polyphenols, since they inhibited hydrolysing enzymes of carbohydrates and intestinal glucose uptake by inhibiting transporters of glucose [63].

Obesity is an independent risk factor for cardiovascular diseases, including hypertension, which means obese patients are twice as prone to coronary heart disease [64]. Recently, the hypotensive effect of *E. dysenterica* leaves reduced the mean arterial pressure in rats in a dose-dependent manner. Those authors related the cardiovascular effects of *E. dysenterica* leaves mainly to vascular action, which seemed to involve the L-type calcium channel blocker, as well as myoendothelial gap-junction signaling [65].

The results indicated that the active compounds of *E. dysenterica* are relevant for preventing obesity and its associated comorbidities, in addition to their potential in the development of new applications and complementary strategies [57]. Among the carotenoids identified in *E. dysenterica* are β-cryptoxanthin and β-carotene. Mice fed a high-fat diet with 0.003% β-cryptoxanthin for 3 weeks were able to revert the hepatic steatosis and insulin resistance, possibly because of its anti-inflammatory action in the liver. The anti-obesity properties of β-cryptoxanthin also suppressed adipogenesis via the activation of retinoic acid receptor (RAR), although the mechanism is still unknown [66,67].

β-carotene inhibited adipogenesis through the production of β-apo-140-carotenal and the inhibition of PPARα, PPARγ and RXR. The ingestion of carotenoids in the diet positively correlated with the concentration of abdominal adipose tissue (a lower correlation was found in the adipose tissue in the buttocks or thighs) for α- and β-carotene, β-cryptoxanthin, cis-lycopene and total carotenoids [65]. The impact of carotenoids in obesity is linked to the fact they are stored, metabolized and bioactive in adipocytes and adipose tissue, thereby limiting the buildup of lipids, inhibiting the differentiation of adipocytes and interfering with nuclear receptors such as RAR, 9-cis retinoic acid receptor (RXR) or PPAR. Lycopene is the most abundant carotenoid in adipose tissue, followed by β-carotene, lutein + zeaxanthin, β-cryptoxanthin and α-carotene [66].

Furthermore, vitamin E inhibits the signaling pathways of mitogen-activated protein kinases (MAPKs), such as extracellular signal-regulated kinase (ERK), c-Jun amino-terminal kinases (JNK) and p38, which are activated by reactive oxygen species (ROS). These pathways are involved in the synthesis of MMPs and pro-inflammatory cytokines and, consequently, the synthesis of collagen. Thus, the reduction of fibrosis in animals treated with vitamin E diminished inflammation and consequently improved sensitivity to insulin [68].

Similarly, low levels of vitamin C are associated with a higher body mass index (BMI) and waist circumference in adults, which can be linked to the effect of vitamin C on leptin expression. It was demonstrated that a lower intake of vitamin C is associated with higher concentrations of leptin in obese women, and in animal models of obesity, it reduced the genic expression of apelin, an adipokine, and its associated comorbidities. Furthermore, vitamin C seems to protect the adipocyte in an obesogenic environment by modulating its interaction with the macrophages and their response to high levels of glucose [69].

Quercetin, also identified in the *E. dysenterica* fruit, shows anti-obesity action for its effect on the metabolism by increasing lipolysis, apoptosis, fattyacid uptake, and inhibiting and reducing lipogenesis, as has already been reported. It also stimulates mitochondrial muscular and hepatic biogenesis and improves glycemia [70].

Evidence suggests that kaempferol reduces the buildup of lipids in adipocytes through the negative regulation of CCAAT-enhancer-binding protein α (Cebpa) without inhibiting new lipogenesis; that is, this compound appears to be a candidate for modulating body weight in obese individuals [71].

The antioxidant and anti-inflammatory properties of ellagic acid are the main contributors to the reduction of adipocyte expansion (hyperplasic and hypertrophic). Studies suggest that supplementation with ellagic acid can reduce expression of the messenger RNA (mRNA) of zinc finger protein 423 (Zfp 423) and aldehyde dehydrogenase 1 family member A1(Aldh1a1), both related to the plasticity of white adipose tissue. It was also observed that ellagic acid increased the genic expression of markers for brown and beige adipocytes, including uncoupling protein 1 (UCP1), PPAR-α, peroxisome proliferator-activated receptor-γ coactivator 1α (PGC1α), cell death-inducing DFFA-like effector A (CIDEa), PR domain-containing 16 (PRDM16), transmembrane protein 26 (TMEM26) and CD137. The mitochondrial biogenesis related to the expression of mitochondrial transcription factor (Tfam) in adipose tissue can improve dyslipidemia induced by obesity, hepatic steatosis and inflammatory responses [72].

The ellagic acid and ellagitannins that remain in the large intestine are metabolized into urolithins present in the microbiota and absorbed by the bloodstream. The ellagitannin-derived metabolites were described as: metabolite A (producer of urolithin-A), metabolite B (producer of urolithin-A, isourolithin-A and urolithin-B) and metabolite 0 (non-producer of urolithins) [72,73]. The production of these metabolites can vary according to body weight and individual gut microbial balance [73].

In a double-blind study, 49 individuals with a BMI > 27 kg/m^2^ consumed daily one (dose 1.0; 160 mg of phenolics/day) or four (dose 2.0; 640 mg of phenolics/day) doses of *Punica granatum* L. (pomegranate) extract or a placebo [72]. The extract and juice of *P. granatum* are known for their concentration of hydrolysable tannins (ellagitannins), including ellagic acid, as well as anthocyanins and other polyphenols [74]. At the end of that study, the hypolipidemic effect was observed only in individuals with metabolite B, along with reductions in total cholesterol, LDLcholesterol, non-apolipoprotein-B cholesterol and LDLcholesterol [72,75].

Among polyphenols, the proanthocyanidins are known for their significative antioxidant capacity, which they achieve by reducing free radicals, protecting against molecular and cellular lesions, improving mitochondrial function and reducing the alteration of hepatic glutathione. A study using proanthocyanidin isolated from grape seed extract demonstrated the ability to diminish metabolic disorders related to obesity, such as hypertension, hyperlipidemia, inflammation and insulin resistance. In an obesogenic rat model, the proanthocyanidin of grape seed extract increased adipocyte hyperplasia and reduced the hypertrophy of the adipose retroperitoneal adipose tissue through inhibition of adipogenesis regulation by PPARγin a sirtuin 1 (Sirt1)-dependent manner. Hyperplasia is associated with the prevention of obesity-related metabolic disorders [75]. In cardiovascular diseases, proanthocyanidins exert their protective effect by inhibiting the oxidation of LDL-cholesteroland binding this molecule to the lectin-like oxidized LDL-1 receptor (LOX-1) involved in the pathogenesis of arteriosclerosis. This phenolic compound also induces vessel relaxation by liberating endothelial nitric oxide and subsequently increasing cyclic guanosine monophosphate in smooth vascular muscle cells [62].

### 3.4. Effects of E. dysenterica on Diarrhea, Antimicrobial Activity and Gut Inflammatory Diseases

The gastrointestinal tract represents the natural interface between gut microflora and host, and the mucosal surface of the gastrointestinal tract is the primary place where microorganisms and ambient antigens interact with the host. Hence, the intestinal microbiota is essential for intestinal development, homeostasis and protection against pathogens and is involved in metabolic reactions, such as fermenting non-digestible food fiber, energy saving and forming short-chain fatty acids (SCFA), biotransforming biliary conjugated acids, degrading oxalate-based complexes and synthesizing vitamins that favoring the development of intestinal microvilli. Furthermore, it plays a fundamental role in the innate and adaptative immunity of the host [76].

The binding of pathogens potentially harmful to the mucosal surface is a crucial step in the development of infections. However, the host has several defense mechanisms that restrict the access of harmful microorganisms: the selective absorption of material from the control exerted by the tight junctions, the liberation of antimicrobial proteins and immunoglobulins in the intestinal lumen, and secretion of mucus. Mucus is secreted by the calciform cells lining the intestine epithelium and is composed mainly of glycosylated proteins, including mucin 2 (MUC2). Villosities and crypts, intraepithelial lymphocytes, Paneth cells, enteroendocrine cells and trunk cells are formed in addition to enterocytes and calciform cells [76].

Paneth and enteroendocrine cells at the base of the crypts specialize in secreting proteinssuch as lysozymes, TNF-α, and natural antimicrobials, such as α-defensins, in the intestinal lumen to limit the buildup of bacterial symbionts in response to bacterial stimulation. In the small intestine, epithelial cells (IECs) secrete antimicrobial lectins, such as the regenerating islet-derived protein 3-gamma (REG3γ), which accumulate in the lumen and impede microorganisms from coming in contact with the epithelium [76].

The cells taking part in the innate phase of the response recognize MAMP antigens, such as LPSs, through pattern recognition receptors (PRRs). Most of them have a high capacity for antigen phagocytosis via the TLR receptors in the intestinal extracellular membrane [76,77]. Activation of these receptors stimulates cascades of intracellular signaling by NOD-like receptors that activate the NF-κB translocation and culminate in the production of several mediators of the immunological response, such as pro-inflammatory cytokines, which stimulate an inflammatory state, necessary to T- and B-lymphocytes that, when exacerbated, provoke intense destruction of tissue. The stimulation of B-lymphocytes induces the production of different types of antibodies. The predominance of immunoglobulin (Ig) A (IgA), IgG or IgE is determined by the stimulus conferred by the T-helper lymphocytes (Th). During the presentation of antigens by the antigen presentation cells (APCs), dendritic and macrophage cells also secrete cytokines that stimulate the lymphocytes Th0, resulting in the generation of a great set of cells, all capable of recognizing a particular pathogen. Th0 cells in the presence of interleukin 12 (IL-12) and interferon-γ (IFN-γ) differentiate into Th1. In addition, the differentiation of Th0 into Th3 and iTreg (induced T regulatory cell) dependsintensely on the presence of transformation growth factor-β(TGF-β) and the expression of the transcription factor of FOXP3 [76,77,78]. These cells start to secrete TGF-β and IL-10 (anti-inflammatory cytokines), which are relevant for controlling the immunological response and the generation of food tolerance. Furthermore, the balance between the Th1 and Th2 responses is an essential factor for avoiding an inappropriate immune response through the T regulator cells (Treg), which can suppress antigen responses and control the immune response [79].

In intestinal dysbiosis, alterations occur in the gut bacteria that hinder homeostasis, linked to the development of intestinal inflammatory diseases, ulcerative colitis, Crohn’s disease and colorectal cancer, but also some extra-intestinal metabolic disorders, such as obesity, diabetes and macro- and microvascular disorders. In intestinal inflammatory diseases, the composition of the intestinal microbiota was altered when compared with the controls, although a uniform pattern of alterations has not yet been observed. The literature reports that pro-inflammatory and pro-oxidative profiles generated in the intestinal lumen and adjacent layers activate immunologic cells (Th1 and Th17), causing damage to tissues, reducing the mucus layer and exacerbating microbial penetration into intestinal tissues. Consequently, there is an increased uptake of microbial antigens and ligands of TLR receptors that perpetuate the immune responses [58].

The use of medicinal plants to treat symptoms of irritable bowel syndrome is widespread because they produce tannins that bind to fluids in the colon, inhibiting the excretory function of diarrhea [80]. In general, secreting diarrhea creates several electrolytic disturbances. In a study with the ethanolic extract of *E. dysenterica*, the authors concluded that the antidiarrhetic effect was not caused by an antisecretory mechanism because they observed electrolytic disturbances inchloride, magnesium and phosphorouslevels [7].

The histopathological analysis of animals treated with aqueous extract and ethanolic extract infusion of *E. dysenterica* at doses of 400 and 800 milligrams·kg^−1^ showed significant alterations in the intestine and hepatic tissue. In animals treated with an infusion of *E. dysenterica* leaves, the presence of intestinal inflammatory infiltrates, partial loss of intestinal villosity and complete alteration of the mucosa with the presence of bleeding was observed, and those that received ethanolic extract showed congestion and alterations in the morphology of the villi. Ulceration and intense inflammation were observed in animals treated with aqueous extract. Serum levels of alanine aminotransferase increased significantly in all treatments, but none rose above reference values. Furthermore, the liver showed congestion and hydropic degeneration, indicating possible toxicity [7].

Intestinal inflammatory diseases, such as Crohn’s disease and ulcerative colitis, are chronic disorders of the gastrointestinal tract characterized by inflammation and epithelial lesions, diarrhea (with or without the presence of blood), abdominal colic and extra-intestinal inflammation, as in the liver [80]. Some of these manifestations were evident in the animals, which were treated mainly by an infusion of *E. dysenterica* leaves, castor oil and loperamide. That observation by those authors is relevant since people use leaf infusions, although it did not have antidiarrheic activity in the study [7].

On the other hand, studies in mice demonstrated that *E. dysenterica* leaf extract protected the gastric mucosa against lesions induced by ethanol and chloric acid by inhibiting the production of chloric acid and presenting antioxidant properties and endogenous sulphydryl (SH)-containing compounds, which induce the elimination of free radicals. In addition, synergism can exist between antioxidant properties and SH-bearing molecules, as the extract can increase the bioavailability of endogenous SH groups [81].

The leaves and fruit of *E. dysenterica* are used for their effects on the gastrointestinal tract. In general, the leaves are used as an antidiarrheic, while the fruit has laxative properties [8].

Fruit pulp in natura and a specific isolated peptide were capable of increasing the intestinal motility of rats. To verify effects, after isolating the peptide from *E. dysenterica* pulp using molecular mass spectrometry, an experimental study on rats had the control group receive a single dose of pulp at 10 mL/kg and peptide at 60 milligrams/kg. The analyses confirmed the capacity of *E. dysenterica* to stimulate intestinal transit by 14.8 and 19%, respectively, without causing diarrhea. Furthermore, histopathological exams of the small intestine of animals treated with pulp in natura and a peptide of *E. dysenterica* did not present alterations. Proteins from *E. dysenterica* fruit could be used to develop new products with laxative properties for treating chronic constipation and irritable bowel syndrome. Such patients generally present a variety of symptoms: abdominal pain and distension, and alternated periods of constipation and diarrhea are the most classic [8,80].

The antidiarrheic activity of *E. dysenterica* leaves was also proven in an animal model of diarrhea induced by castor oil. The ethanolic extract at a dose of 400 mg/kg and loperamide at 2 mg/kg inhibited the intestinal transit significantly in rats by 24 and 37%, respectively. However, the aqueous extract prepared by the infusion of leaves at a dose of 800 mg/kg did not affect intestinal motility. Antidiarrheic activity was observed in plants with compounds such as tannins, alkaloids, saponins, flavonoids, steroids and terpenoids [7]. The condensed tannins identified in the leaf extract of *E. dysenterica*, predominantly procyanidin and prodelphinidin, were mainly responsible for the gastroprotective effect. These compounds seem to bind to the epithelial mucin in the gastric mucosa to form a protective lining against harmful agents [82].

The essential oil of *E. dysenterica* leaves (comprised of *cis*-b-ocimene, (*E*)-caryophyllene, caryophyllene oxide, a-humulene, linalool and trans-b-ocimene) significantly reduced diarrhea. The antidiarrheic effect of the essential oil seems to be related to its capacity to inhibit intestinal secretion and or increase intestinal absorption. Inhibition of gastrointestinal motility was also not involved in its antidiarrheic effect, which means more studies are needed to clarify the molecular mechanism [83].

Tannins, flavonoids and phenolic compounds, the main constituents of *E. dysenterica* leaves, are responsible for preventing diseases associated with oxidative damage to membranes, proteins and DNA. They also have antimutagenic, anticarcinogenic and anti-aging properties [84]. Moreover, in the fruit pulp, bioactive compounds such as proanthocyanidins, flavonoids (quercetin derivatives, kaempferol, phenolic acid, ellagic acid) and carotenoids (α-carotene, β-carotene, lycopene) were found [85].

The phenolic substances inserted in the diet, mainly fromfruit and vegetables, produced prebiotic effects and antimicrobial action against diseases related to dysbiosis [86]. In vitro studies suggested that polyphenols modulate microbiota, thereby inhibiting pathogenic bacteria, such as *Helicobacter pylori*, and favoring the growth of *Lactobacillus* and *Bifidobacteria*; thus, the prebiotic activities of polyphenols are a leading mechanism that benefits human health [86].

Astudy of C57BL/6 micesuggested that the dietary supplementation of 30 mg/kg quercetin exerts therapeutic effects on colitis induced by *Citrobacter rodentium* by suppressing pro-inflammatory cytokines (e.g., interleukin 17 (IL-17), TNF-α and IL-6) and promoting the production of IL-10 in the colon tissues. Quercetin supplementation also modified the gut microbiota, significantly increasing the populations of *Bacteroides*, *Bifidobacterium*, *Lactobacillus* and reducing those of *Fusobacterium* and *Enterococcus*. These results suggest that quercetin directly stimulated the immune system to reduce inflammation and restore the intestinal microbiota balance; therefore, quercetin is effective for reducing the pathologic effects of colitis in animalsinduced by *C. rodentium* [87].

Kaempferol seemed to have a protective effect through mechanisms related to the mechanical and biological intestinal barrier. There was increased expression of proteins TJ ZO-1 and occludin, as well as of proteins of the butyrate receptor GPR109A and transporter SLC58A. Plasma levels of alanine aminotransferase (ALT) and aspartate aminotransferase (AST) were reduced in an animal model of hepatic alcoholic lesions treated with kaempferol at low, medium and high doses: 25, 50, 100 mg/kg. Alcohol affectedthe composition and functioning of the gut microbiota in two ways: directly, by increasing endotoxemia and the permeability of the intestinal barrierto macromolecules, and indirectly when their metabolites reached the bloodstream. That cause is well-established. The mechanical barrier is controlled by specific proteins and intercellular adhesion pre-proof complexes, including occludins, zonula occludens 1, and adherens junction complexes. Among these, tight junction proteins are the major determinants of intestinal physical barriers [88].

Evidence suggests that the consumption of food rich in ellagic acid can attenuate metabolic disorders associated with the gut microflora and its production of urolithin [87]. As described before, ellagic acid and ellagitannins that remain in the large intestine after being consumed are metabolized into urolithins by the microbiota and absorbed by the bloodstream. Recently, the metabolites of ellagitannins were described as metabolite A (producer only of urolithin-A), metabolite B (producer of urolithin-A, plus isourolithin-A and urolithin-B) and metabolite 0 (non-producer of these urolithins) [71,72]. However, gut microbial imbalances favor the growth of bacteria capable of producing isourolithin-A or urolithin-B instead of urolithin-A. Species of *Gordonibacter* can transform ellagic acid into different urolithins in pure culture and positively correlate with urolithin-A in feces and urine, while the occurrence of isourolithin-A and urolithin-B negatively correlates with the fecal concentration of *Gordonibacter* spp. [72].

Some liposoluble vitamins in the pulp of *E. dysenterica*, such as vitamin A, are involved in the regulation of gut homeostasis, epithelial integrity, innate and adaptative immune systems, and gut microbiota. It is a fact that a high number of immune cells and cells of the gastrointestinal tract express receptors of vitamins D and A. A deficiency of these vitamins in animals reduced microbial diversity and increased potentially pathogenicbacteria, such as *Proteobacteria phylum*. Patients with intestinal inflammatory disease in pre-clinical studies corroborated these findings. Studies in vitroalso showed that vitamins A and D are involved in the regulation of the intestinal barrier function through the expression of tight junction molecules [77].

Supplementation with β-carotene raised the production of IgA and immune tissues associated with the intestine; thus, carotenoids play an essential role in cellular function, such as regulating the immune system and activatingthe responses of macrophages, T cells and Treg cells. However, microbiota use of carotenoids is not yet demonstrated [89].

The natural antioxidants vitamins A, C and E regulate gut microbiota composition by eliminating free radicals and enhancing immune responses. In an experimental study, mice received a diet enriched in vitamin E, selenium and retinoic acid. The antioxidants were able to attenuate the mucosa inflammation by remodeling the intestinal microbiota, increasing the percentage of *Bacteroidetes* and reducing *Firmicutes*. Furthermore, in another study, a lower proportion of *Firmicutes* compared with *Bacteroidetes* in mice treated with high doses of vitamin E (0.18 mg/20 g of body weight) in comparison with the control and low-dose treatment (0.06 mg/20 g of body weight) was observed. Research on pregnant women corroborated the experimental results; indeed, a higher intake of vitamin E diminished the number of *Proteobacteria* and *Firmicutes* and increased *Bacteroidetes* in their gut microbiota. The intake of the soluble antioxidant vitamin C increased the elimination of free radicals and the count of *Lactobacillus* and *Bifidobacterium* and decreased the *E. coli* count in the intestinal microbiota [90].

Overall, dietary polyphenols showed beneficial effects on intestinal microbiota; however, specific studies are needed to demonstrate the effects of the polyphenols already identified in *E. dysenterica* on the modulation of populations of intestinal microbiota. The main effects of *E. dysenterica* on obesity and intestinal inflammatory diseases are summarized in Table 4.

## 4. Conclusions

*Eugenia dysenterica*contains phenolic compounds, carotenoids, ascorbic acid, β-carotene, lycopene, α-, β-, γ- and δ-tocopherol, folates and tannins that show potential antioxidant action and inhibitory activity on enzymes involved in the metabolism of carbohydrates. It, therefore, has a role in preventing the development of obesity and diabetes mellitus type 2, fighting hyperglycemia, hyperlipidemia and reducing hypertension. In addition, *E. dysenterica* improved intestinal motility, enhanced protection of the gastric mucosa against aggressor agents and prevented inflammation. The phenolic compounds in *E. dysenterica* fruit and leaves had the highest nutraceutical potential. However, studies are necessary to identify the mechanism by which *E. dysenterica* nutraceutical compounds act on such pathological processes to support future research.

## Figures and Tables

**Table 1:** Nutritional value of the macronutrients and fibers of *E. dysenterica* DC. per 100 g, and energy of pulp and seeds [18,19].

Components	Pulp	Seeds
Proteins (g)	1.140.63	0.59
Carbohydrates (g)	6.33	14.85
Lipids (g)	0.05	0.26
Fibers (g)		1.05
Total energetic value (kcal)	30.33	64.10

**Table 2:** Main phytochemical compounds and minerals (mg) in *E. dysenterica* DC. per 100 g of fruit, pulp and seeds [22,24].

*Eugenia dysenterica* DC.			
Phytochemical Compounds	Pulp	Seeds	Leaves
Ascorbic acid Folates Carotenoid β-carotene Lycopene Tannins α-, β-, γ- e δ-Tocopherol	24.53 mg 25.74 μg 0.77 mg − − − −	− − − + + − −	− + − + + + +
**Minerals**	**Pulp**	**Seeds**	**Leaves**
Calcium Iron Zinc Phosphorous Potassium Magnesium Sulphur Boron Copper Manganese	8.0 mg 0.02 mg − − − − − − − −	− − − − − − − − − −	0.84% 145 ppm 21.40 ppm 0.14% 1.20% 0.28% 0.06% 32.50 ppm 15 ppm 163 ppm

Subtitle: −: not quantified or absent, +: present and not quantified.

**Table 3:** Bioactive compounds in *E. dysenterica* DC. [25,26,27,28,29,30].

*Eugenia dysenterica* DC.		
Bioactive Compounds	Pulp	Leaves
Quercetin Kaempferol Ellagic acid Ellagitannis Proanthocianidins Phenolic acids Flavonoids Anthocyanins Organic acids Polyphenols γ-Cadinene δ-Cadinene	+ + + + + + + + + + − −	− − − − + − − − − + + +

Subtitle: −: not quantified or absent, +: present and not quantified.

**Table 4:** Main effects of *Eugenia dysenterica* DC. on obesity and intestinal inflammatory diseases.

Plant Part	Host	Treatments	Main Effects	Reference
Leaf aqueous extract	Gastric lesion model induced by acidified ethanol	6 groups of rats that had fasted for 24 h were given oral saline solution, carbenoxolone or *E. dysentherica* leaf aqueous extract (100, 300, 550, 1000 mg/kg); 50 min after treatments, all received oral HCl 0.3 M/ethanol at 60%. Antioxidant activity of the extract determined in vitro.	↑ protection of gastric mucosaagainst lesions induced by ethanol/HCl-c, ↓ production of HCl-c, ↓ free radicals, ↑ protective liningagainstharmful agents (aqueous extract 550 and 1000 mg/kg).	[82]
Pulp *in natura* and peptide	Male rats	Positive and negative control groups received oral castor oil and water at the dose of 10 mL/kg. Control groups received pulp at 10 mL/kg and peptideat 60 mg/kg.	↑ intestinal transit by 14.8% (pulp *in natura*) and 19% (peptide) without causing diarrhea.	[8]
Aqueous infusion and ethanolic extract	Animal model of diarrhea induced by castor oil	Forty rats were separated into five groups of eight animals. The positive control group received loperamide orally at a dose of 2 mg/kg, and the negative group received 1 mL of water; test groups received aqueous extract at doses of 800 mg/kg or ethanolic extract at doses of 400 mg/kg.	↓ intestinal transit by 24%, ↑ serum chloride levels, ↓ serum phosphorus and magnesium levels, ↑ alanine aminotransferase levels (ethanolic extract).	[7]
Pulp Extract	Male mice C57BL/6J, obesity model induced by high fat diet and sucrose	Group 1: A chow-fed group + water administrated by gavage Group 2: high-fat high-sucrose diet + water administered by gavage Group 3: high-fat high-sucrose diet + phenolic-rich extract from *E. dysentherica* by gavage (7 mg GAE/kg body weight) Group 4: high-fat high-sucrose diet + phenolic-rich extract from *E. dysentherica* by gavage (14 mg GAE/kg body weight).	↓ body weight gain, ↓ increase in retroperitoneal, epididymal and brown adipocyte deposits, ↓ fecal lipids, ↓ levels of plasma and hepatic triacylglycerols, ↑ plasma (pulp extract).	[26,84]
Pulp extract	Animal model for obesity study	After 6 weeks of feeding with high-fat high-sucrose (HFS) diet or chow diets, mice in the HFS dietary group were randomlydivided into three groups: Group 1: HFS + water given by gavage Group 2: HFS + phenolic-rich extract from *E. dysentherica*given by gavage (7 mg GAE/kg body weight) Group 3: HFS + phenolic-rich extract from *E. dysentherica* given by gavage (14 mg GAE/kg body weight) Group 4: A chow-fed group + water administered by gavage.	↓ adipocyte size, ↓ hyperglycemia and dyslipidemia, ↓ serum levels of NEFA and LDL-cholesterol, ↓ aminotransferase activity, ↓ fasting hyperglycemia, ↓glucose intolerance, ↓ pyruvate carboxylase mRNA (pulp extract).	[57,88]
Juices from Brazilian native fruit	Healthy volunteer individuals	Each meal consisted of ca. 25 g of available carbohydrate as white bread (corresponding to one unit ca. 50 g) and 300 mL of water (control), or clarified fruit juices, given after 10–12 h of fasting. All underwent tests with water (control) and clarified juices at 7-day intervals in between.	↓ postprandial glucose, ↓ oxidative stress (juice of pulp)	[62]
Aqueous leaf extract	Animal model for cardiovascular study	Procedures in rats under anesthesia. Direct measure of arterial pressure and intravenousadministration of extract or drugs through adequate inserted polyetene catheters in the femoral artery and vein, respectively.	↓ mean arterial pressure, type-L calcium channel blockade, as well as myoendothelial gap-junction signaling (aqueous leaf extract)	[63]
